# Cost-effectiveness of adding rituximab to splenectomy and romiplostim for treating steroid-resistant idiopathic thrombocytopenic purpura in adults

**DOI:** 10.1186/s12913-015-0681-y

**Published:** 2015-01-22

**Authors:** Kayoko Kikuchi, Yoshitaka Miyakawa, Shunya Ikeda, Yuji Sato, Toru Takebayashi

**Affiliations:** Center for Clinical Research, Keio University School of Medicine, 35 Shinanomachi, Shinjuku-ku, Tokyo Japan; Department of General Internal Medicine, Saitama Medical University Hospital, 38 Morohongo Moroyamamachi, Irimagun, Saitama Japan; Department of Pharmaceutical Sciences, School of Pharmacy, International University of Health and Welfare, 2600-1 Kitakanemaru, Ohtawara City, Tochigi Japan; Department of Preventive Medicine and Public Health, Keio University School of Medicine, 35 Shinanomachi, Shinjuku-ku, Tokyo Japan

**Keywords:** ITP, Splenectomy, Romiplostim, Rituximab, Cost-effectiveness

## Abstract

**Background:**

Idiopathic thrombocytopenic purpura (ITP) is an autoimmune disease in which the platelet count falls to <100 × 10^9^/L. Corticosteroids are recommended as the first-line treatment, splenectomy is recommended as the second-line treatment, and thrombopoietin receptor agonists (TPO-RAs) and rituximab are recommended as the third-line treatments for ITP in Japanese ITP treatment guidelines. However, in Japan, rituximab is not eligible for reimbursement for the treatment of ITP. The cost-effectiveness of ITP treatment has not been investigated in Japan. Therefore, in this study, the cost-effectiveness of adding rituximab treatment to the existing treatments indicated for ITP in Japan, namely splenectomy and the TPO-RA romiplostim, was investigated based on the scenario that rituximab is eligible for reimbursement in Japan as a treatment for ITP.

**Methods:**

The efficacy endpoint was set as the number of years with a platelet count ≥30 × 10^9^/L. The analysis was conducted from the healthcare payer’s perspective. If the first treatment is ineffective or relapse occurs, then the patient is given the following treatment. The analyzed treatment order consisted of three patterns: splenectomy-romiplostim (sequence 1), splenectomy-romiplostim-rituximab (sequence 2), and splenectomy-rituximab-romiplostim (sequence 3). A Markov model was built for ITP, and the analysis period was set as 2 years. The discount rate was an annual rate of 2%.

Sensitivity analyses of the efficacy of splenectomy, romiplostim, and rituximab; treatment cost; and romiplostim dose were performed.

**Results:**

The expected costs per patient over a 2-year period for sequences 1, 2, and 3 were USD 40,980, USD 39,822, and USD 33,551, respectively. The expected years with a platelet count ≥30 × 10^9^/L for the three sequences were 1.75, 1.79, and 1.78 years, respectively. The sensitivity analyses illustrated that the results of the base case analysis were robust.

**Conclusions:**

Adding rituximab to standard treatment for ITP (sequences 2–3) is less costly and marginally more effective than standard therapy in adults. According to the study results, if rituximab is reimbursed for the treatment of ITP in Japan, medical expenses are expected to decline.

## Background

Idiopathic thrombocytopenic purpura (ITP) is an autoimmune disease in which the platelet count falls to <100 × 10^9^/L. There are approximately 20,000 patients with this disorder in Japan, and it is reported that approximately 3,000 people develop this disorder every year.

The pathology of the disorder involves destruction of autoantibody-presenting platelets by the spleen and liver, and insufficient hematopoiesis of megakaryocytes is also observed because of the relative shortage of the megakaryocyte hematopoietic factor thrombopoietin. ITP is classified based on the length of time from the diagnosis as follows: newly diagnosed, <3 months; persistent, between 3 and 12 months; and chronic, >12 months. Ninety percent of patients with adult-onset ITP have chronic disease, and the male-to-female ratio is 1:2. Approximately 10% of all patients with ITP do not respond to standard treatment, and cases in which the platelet count falls to <30 × 10^9^/L are considered intractable. The mortality rate of patients with ITP is 4.2-fold higher than that of healthy individuals [1]. However, opportunities to select immunosuppressive agents have declined because of the current availability of rituximab and thrombopoietin receptor agonists (TPO-RAs), and it is possible that the risk of death, even if the platelet count is <30 × 10^9^/L, is no longer 4.2-fold higher than that of healthy individuals.

The Japanese ITP treatment guidelines are as follows. Patients with ITP who are infected with *Helicobacter pylori* first receive *H. pylori* eradication therapy [2], which recovers the platelet count to 100 × 10^9^/L in approximately half of all patients. However, when *H. pylori* eradication therapy is ineffective or patients are not infected with *H. pylori*, corticosteroid treatment is the first-line treatment option, and this treatment is highly effective. However, only 10%–20% of all patients can discontinue corticosteroid administration, and most patients require long-term corticosteroid treatment in Japan. This long-term corticosteroid treatment is significantly problematic clinically because it causes side effects such as diabetes, hypertension, peptic ulcers, and immunodeficiency.

When corticosteroids are ineffective or there are problems with tolerability, a splenectomy is the second-line treatment option. The efficacy rate of splenectomy is reported to be approximately 60%, although relapses are also reported [3]. The drawbacks of splenectomy are risks such as perioperative complications experienced by 10% of subjects, the mortality rate of 1% associated with the laparotomic portion of the procedure, the mortality rate of 0.2% associated with laparoscopic surgery, and severe infection because of postoperative immunodeficiency [1,3].

Patients for whom the splenectomy was ineffective or who are not candidates for surgery can receive third-line treatment with the anabolic steroid danazol, the immunosuppressants azathioprine and cyclosporine, the anticancer agents vincristine and cyclophosphamide, the TPO-RAs romiplostim and eltrombopag, and the anti-CD20 monoclonal antibody rituximab. Among the third-line treatment options, only TPO-RAs are covered by health insurance in Japan, and romiplostim and eltrombopag were approved after 2011. TPO-RAs are effective in approximately 60%–90% of intractable cases; however, once treatment is discontinued, the platelet count decreases to the pre-treatment level within 2 weeks [4-6]. This demands long-term treatment, which places financial pressure on the patient because of high drug costs. It was recently reported that some patients do not experience a relapse after TPO-RA discontinuation. However, further investigation via a larger-scale, longer-term observational study is required because these data were obtained from a short-term observational study involving a small number of subjects.

By contrast, another third-line treatment option, rituximab, basically requires only four doses administered at weekly intervals, and the patient can expect a radical cure; thus, this drug is less expensive than TPO-RAs. In a systematic review of the curative effect of rituximab targeting approximately 300 patients with ITP, it was reported that the platelet count exceeded 50 × 10^9^/L in 62.5% of patients [7]. The median response duration is limited; after 5 years, 20%–25% of patients have sufficient platelet counts [7]. Rituximab is described as a second-line treatment option for intractable ITP in the American guidelines [5]. However, in Japan, there are few records of the use of rituximab for treating ITP; therefore, it is not indicated for ITP, and it is currently used off-label [8]. ITP was specified as an intractable disease in 1974 in Japan, and some patients are eligible for public financial support from the national and prefectural governments. However, drugs such as rituximab that are not indicated for ITP are neither eligible for public financial assistance nor are they covered by insurance. The reality is that these drugs are used off-label to save the lives of patients with intractable ITP. At this juncture, clinical trials are currently underway under the assumption that rituximab is eligible for reimbursement in Japan as a treatment for ITP.

Recently, there has been increased interest in Japan in the evaluation of medical economy, with investigations into the introduction of the Health Technology Assessment (HTA) by the Central Social Insurance Medical Council. However, the cost-effectiveness of ITP treatment has not been investigated in Japan. There are reports overseas that investigated the cost-effectiveness of TPO-RA with standard ITP treatments, including rituximab [9-11], but there are no reports that investigated the cost-effectiveness of splenectomy followed by romiplostim (sequence 1) and splenectomy-romiplostim plus rituximab (sequences 2–3).

In this study, based on the scenario that rituximab is eligible for reimbursement in Japan as a treatment for ITP, we investigated the cost-effectiveness of adding rituximab to splenectomy and the TPO-RA romiplostim.

## Methods

The aim of this study was to clarify the cost-effectiveness of adding rituximab to conventional treatments for ITP in adults, namely splenectomy and romiplostim. A Markov model was built for ITP, and the analysis period was set at 2 years. The efficacy endpoint was the number of years with a platelet count ≥30 × 10^9^/L: the level required to prevent life-threatening blood loss. The costs were set as the direct medical costs. The analysis was conducted from the healthcare payer’s perspective. All costs are shown in US dollars.

### Treatment option

A typical adult patient with intractable ITP for whom the first treatment option of corticosteroids is ineffective was set as a target Japanese woman of 50 years of age, 60 kg in weight, and 160 cm in height.

**Figure 1:**
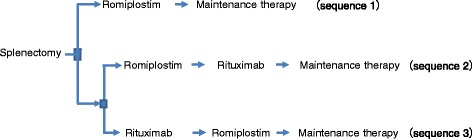
**Analyzed treatment order for idiopathic thrombocytopenic purpura.**

In accordance with the Japanese ITP treatment guidelines, a patient with intractable ITP who is non-responsive to corticosteroids undergoes splenectomy. If the splenectomy is ineffective or relapse occurs, then the patient is switched to romiplostim or rituximab treatment. Furthermore, if romiplostim is ineffective, then the patient is switched to rituximab, and if rituximab is ineffective, the patient is then switched to romiplostim. If splenectomy, romiplostim, and rituximab are all ineffective or relapse occurs, then the patient is switched to maintenance therapy using corticosteroids. Because this study targeted patients with intractable ITP, the analysis started with the second-line treatment option of splenectomy. Therefore, the analyzed treatment order consisted of three patterns as follows: splenectomy-romiplostim (sequence 1), splenectomy-romiplostim-rituximab (sequence 2), and splenectomy-rituximab-romiplostim (sequence 3) (Figure 1).

Prior to undergoing splenectomy, the patient is administered five doses of 0.4 g/kg IVIG as an outpatient therapy to adequately increase the platelet count, and the splenectomy is performed during a 7-day hospitalization.

At present, two TPO-RAs are indicated for treating ITP in Japan, and for this study, we conducted a simulation using one of those products, romiplostim. Romiplostim is an injectable treatment that is administered at 1–10 μg/kg body weight per week. The dose will be titrated individually, and in some patients, it is possible to use a much lower dose than the licensed dose. The dose in this study was 4 μg/kg based on the results of long-term continuation tests in Japanese patients; therefore, a dose of 240 μg was calculated for a 60-kg patient. One vial of romiplostim contains 250 μg of the drug. Therefore, one vial is used per dose, assuming that the remainder would be discarded [12].

A single dose of rituximab was set at 375 mg/m^2^, and it was assumed that a total of four doses would be administered at weekly intervals. The initial dose of rituximab requires the patient to stay in the hospital overnight, whereas the remaining three doses are administered in the outpatient setting.

Assuming that sepsis would occur during the splenectomy and rituximab treatment period as well as after treatment, any affected patient would be hospitalized for treatment [7,13]. Concerning the romiplostim treatment period, pulmonary embolism was assumed as the adverse event, and any affected patient would be hospitalized for treatment [14].

Emergency treatment for exacerbation of ITP symptoms such as a rapid decline in the platelet count and hemorrhage involved hospitalization and IVIG administration.

If the platelet count does not reach or exceed 30 × 10^9^/L after splenectomy, romiplostim, and rituximab treatment, corticosteroids (20 mg/day) was administered as a maintenance therapy.

### Model design

**Figure 2:**
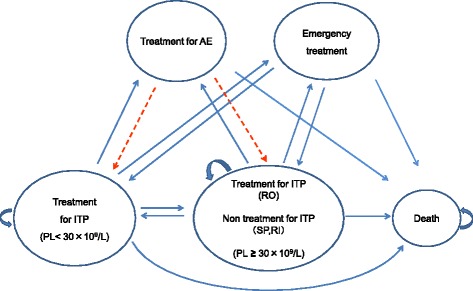
**Structure of the model.** AE: adverse event, PL: platelet, SP: splenectomy, RO: romiplostim, RI: rituximab. The model is composed of six states. Each cycle was a 28-day period. The cycle starts from the idiopathic thrombocytopenic purpura (ITP) treatment state (PL <30 × 10^9^/L) and moves to the next state after 28 days. However, if PL remains <30 × 10^9^/L after the treatment, the cycle remains at the ITP treatment state. The broken line shows the flow of patients treated with splenectomy and rituximab, i.e., patients who received treatments other than romiplostim.

We created a model of the typical clinical course of ITP using a Markov model based on the advice of ITP specialists (Figure 2). One cycle consisted of 4 weeks based on a report that investigated the cost-effectiveness of romiplostim [11]. The response time was set in reference to the American guidelines for ITP [5]. The transfer rates to each of the disease states were summarized based on academic reports and specialists’ opinions. “Effective” was defined as a platelet count ≥30 × 10^9^/L after treatment. “Relapse” was defined as a platelet count ≥30 × 10^9^/L after treatment that subsequently declined to 30 × 10^9^/L.

The model was created based on the following assumptions. If splenectomy and rituximab treatment are effective, then there is no subsequent treatment. If the treatment is ineffective or the patient relapses, the patient is then switched to the following treatment. Assuming that the adverse event during the perioperative period of splenectomy and rituximab administration or after these treatments was sepsis, the patient would receive the treatment for sepsis for 4 weeks, after which the original treatment would be resumed. If romiplostim treatment is effective, then the patient would continue this treatment. If the treatment is ineffective, the patient would then be switched to the following treatment. If pulmonary embolism occurred during romiplostim treatment, the patient would discontinue treatment with romiplostim and receive 4 weeks of treatment for the pulmonary embolism. If emergency treatment is required because of exacerbation of ITP symptoms in any of the treatment groups, the patient would be hospitalized for 4 weeks and administered IVIG, after which the original treatment would be resumed. If no effect is observed after treatment with splenectomy, romiplostim, and rituximab or if relapse occurs, the patient would then receive maintenance therapy with corticosteroids. However, because this study targeted intractable ITP that was non-responsive to corticosteroids, the platelet count would remain at <30 × 10^9^/L even after corticosteroid administration. The adverse event assumed to occur during corticosteroid maintenance therapy was diabetes, and if diabetes occurred, corticosteroid maintenance therapy would be continued while also administering diabetes treatment.

### Model inputs

The efficacy rates, relapse rates, and incidence of adverse events obtained from the literature are presented in Table 1. These rates were used as the transfer rates between the states.

**Table 1:** **Probability parameters**

**Events**	**Splenectomy**	**Romiplostim**	**Rituximab**	**Corticosteroids**
Efficacy rates	76.1% [15]	84.1% [14]	71.6% [16,17]	―
Relapse rates	0.45% [3]	0%*	0.88% [18]	―
Adverse event rates	0.44% [13]	0.58% [14]	0.24% [7]	11% [19]
Emergency treatment rates (PL < 30 × 10^9^/L)	14.4% [20]	14.4% [20]	14.4% [20]	26.8% [20]
Emergency treatment rates (PL ≥ 30 × 10^9^/L)	0.4% [20]	0.4% [20]	0.4% [20]	―
Mortality rate (PL < 30 × 10^9^/L)	GR (%) × 4.2 + 0.02%^†^[1,3,21]	GR (%) × 4.2 [1,21]	GR (%) × 4.2 [1,21]	GR (%) × 4.2 [1,21]
Mortality rate (PL ≥ 30 × 10^9^/L)	GR (%) × 1.8 [1,21]	GR (%) × 1.8 [1,21]	GR (%) × 1.8 [1,21]	GR (%) × 1.8 [1,21]

PL: platelet, GR: General mortality rate. References are given in brackets.

*If romiplostim treatment is effective, then relapse would not occur.

^†^A mortality rate of 0.02% was added when splenectomy was conducted.

The annual mortality rate was calculated by multiplying the mortality rate of the target patients (Japanese women aged 50 years) by the risk of mortality based on reports indicating that the mortality risk of patients with ITP and a platelet count <30 × 10^9^/L is 4.2-fold higher than that of healthy subjects, compared to 1.8-fold higher than that of patients with a platelet count ≥30 × 10^9^/L [1]. The mortality rate of 0.02% associated with surgery was added when splenectomy was conducted.

The costs used for the analysis are shown in Table 2. The costs were set as the direct medical costs from the healthcare payer’s perspective. All costs are shown in US dollars. The National Health Insurance drug prices revised on April 1, 2012 were used to determine the cost of each drug. The items and number of standard tests and treatments were determined based on the ITP Treatment Guidelines and the opinions of ITP specialists. The cost for one cycle (28 days) was summed based on the hospital and treatment fee schedule. Currency conversions were calculated using the exchange rate as of November 2013 (1 USD = JPY100).

**Table 2:** **Cost parameters**

**Item**		**Cost (USD/28 days)**
Treatment for ITP	Splenectomy	19,124
	Romiplostim	2,806
	Rituximab	12,582
	Corticosteroids	91
Death	-	0
Treatment for AE	Sepsis	4,329
	Pulmonary embolus	4,101
	Diabetes	243
Emergency treatment	IVIG	15,533

AE: adverse event.

The discount rate was an annual rate of 2% based on the guidelines for the economic evaluation of healthcare technologies in Japan [22].

### Cost-effectiveness

The expected costs required for a patient to receive treatment for 2 years for sequences 1–3 were calculated based on the transfer rate to each of the disease states obtained from the literature and the cost of each component (Table 2). The efficacy endpoint was set as the number of years with a platelet count ≥30 × 10^9^/L. The cost-effectiveness ratio was set as 2-year expected cost/years with a platelet count ≥30 × 10^9^/L. The cost required to maintain the platelet count at ≥30 × 10^9^/L for 1 year. We compared the cost-effectiveness ratio for sequences 1, 2, and 3.

### Sensitivity analyses

Unless the cost-effectiveness ratio of sequence 2 is larger than that of sequence 1, sequence 2 is cost-effective compared to that of sequence 1. The relationship is considered the same between sequences 1 and 3; therefore, a sensitivity analysis was conducted focusing on the differences between these cost-effectiveness ratios.

The efficacy of splenectomy, romiplostim, and rituximab varied by 50% below the base case analysis in sensitivity analyses. Concerning the costs, the costs of splenectomy, rituximab, and emergency treatment varied by 50% above and below the base case analysis in sensitivity analyses.

Furthermore, in the sensitivity analyses, the romiplostim dose was set as two vials per dose instead of one vial as set in the base case analysis because even slight changes in body weight would lead to changes in the number of vials if the remainder of the drug was assumed to be discarded.

## Results

### Cost-effectiveness

The expected costs per patient over a 2-year period are shown in Table 3. The expected costs per patient over a 2-year period for sequences 1, 2, and 3 were USD 40,980, USD 39,822, and USD 33,551, respectively.

**Table 3:** **Result of base case analysis**

**Treatment order**	**2-year expected cost (USD)**	**Expected period of the PL ≥ 30 × 10** ^**9**^ /L (years)*	**Deaths** ^**†**^	**Manifesting AE** ^**†,‡,§**^	**Emergency treatment** ^**†**^	**Cost-effectiveness ratio** ^**||**^
Sequence 1	40,980	1.75	87.02	224.47	140.96	23,438
Sequence 2	39,822	1.79	85.44	114.78	110.66	22,280
Sequence 3	33,551	1.78	85.65	106	128.83	18,826

PL: platelet, AE: adverse event.

*The period was calculated as N_S_ · P/N_T_, where N_S_, P, and N_T_ denote the average number of people in the PL ≥ 30 × 10^9^/L states in the simulated population, the duration of simulation (2 years), and the total number of people simulated, respectively (10,000).

^†^The number of patients among 10,000 people.

^‡^Assuming sepsis would occur during the splenectomy and rituximab treatment period as well as after treatment. Pulmonary embolism was assumed to be the adverse event for romiplostim treatment.

§Using these AE incidence rates as the rates of transition among the states of the Markov model to a state of “treatment for AE,” we calculated the number of people who developed adverse events in 2 years in a 10,000-person population.

|| Two-year expected cost (USD)/period of PL ≥ 30 × 10^9^/L (years).

**Figure 3:**
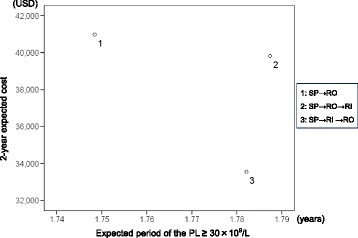
**Relationship between 2-year expected cost and period of the PL ≥ 30 × 10** ^**9**^ **/L.** PL: platelet, SP: splenectomy, RO: romiplostim, RI: rituximab.

The cost-effectiveness ratio or 2-year expected cost/years with a platelet count ≥30 × 10^9^/L, was USD 23,438 for sequence 1, USD 22,280 for sequence 2, and USD 18,826 for sequence 3 (Table 3, Figure 3).

The results of the base case analysis illustrated that the addition of rituximab (sequences 2–3) was cost-effective compared to that of splenectomy-romiplostim (sequence 1).

### Sensitivity analyses

**Figure 4:**
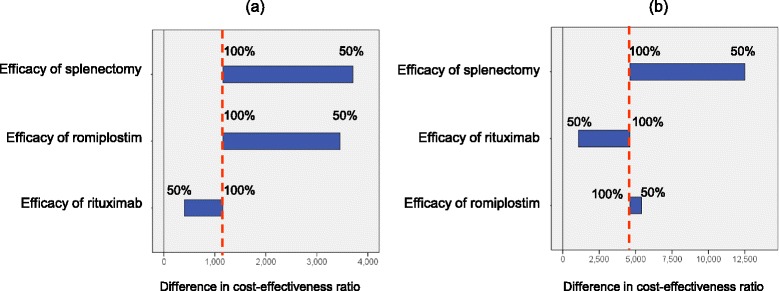
**Results of sensitivity analyses on efficacy rates.** The efficacy of splenectomy, romiplostim, and rituximab varied by 50% below the base case analysis. Broken lines denote the results of the difference in the cost-effectiveness ratio in the base case analysis. **(a)** Difference in the cost-effectiveness ratios between sequences 1 and 2. **(b)** Difference in cost-effectiveness ratios between sequences 1 and 3.

Table 4 presents the differences in cost-effectiveness ratios between sequences 1 and 2 and between sequences 1 and 3. The sensitivity analysis was conducted using the differences in these cost-effectiveness ratios. The efficacy of splenectomy had the most significant effect in the sensitivity analyses. When the efficacy of splenectomy varied by 50% below the base case analysis, the differences between the cost-effectiveness ratios of sequences 1 and 2 and between sequences 1 and 3 increased from USD 1,158 to USD 3,707 and from USD 4,612 to USD 12,503, respectively (Figure 4). When the rituximab efficacy varied by 50% below the base case analysis, the differences between the cost-effectiveness ratios of sequences 1 and 2 and between sequences 1 and 3 decreased from USD 1,158 to USD 406 and from USD 4,612 to USD 1,079, respectively, but the difference did not fall below 0 (Figure 4).

**Table 4:** **Cost-effectiveness ratio**

**Treatment order**	**2-year expected cost (USD)**	**Expected period of the PL ≥ 30 × 10** ^**9**^ **/L (years)**	Cost-effectiveness ratio*	**Difference between cost-effectiveness ratio**
Sequence 1	40,980	1.75	23,438	ー
Sequence 2	39,822	1.79	22,280	1,158^†^
Sequence 3	33,551	1.78	18,826	4,612^‡^

PL: platelet.

* Two-year expected cost (USD)/period of PL ≥30 × 10^9^/L (years).

^†^Differences in the cost-effectiveness ratios between sequences 1 and 2.

^‡^Differences in the cost-effectiveness ratios between sequences 1 and 3.

The costs of the treatments did not have great influence on the differences between the cost-effectiveness ratios.

**Figure 5:**
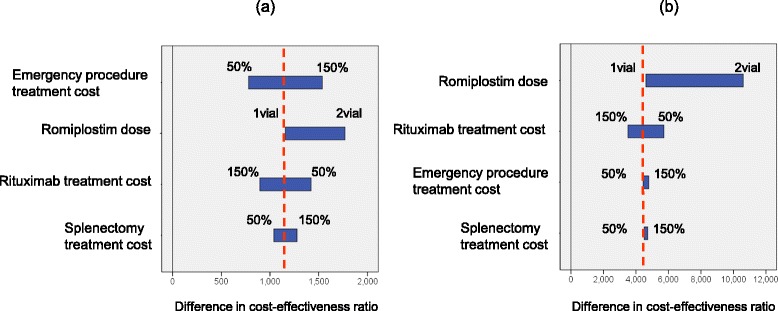
**Sensitivity analysis of cost and the romiplostim dose.** The treatment costs varied by 50% above and below the base case analysis, and the romiplostim dose varied from one to two vials. Broken lines denote the results of the difference in the cost-effectiveness ratios in the base case analysis. **(a)** Difference in the cost-effectiveness ratios between sequences 1 and 2. **(b)** Difference in the cost-effectiveness ratios between sequences 1 and 3.

When the romiplostim dose was set as two vials per dose, the difference between the cost-effectiveness ratios of sequences 1 and 3 increased from USD 4,612 to USD 10,603 (Figure 5).

The sensitivity analyses revealed that the results of the base case analysis were robust.

## Discussion

This study created an independent model in which the standard platelet count used for the endpoint was set at 30 × 10^9^/L, with states including hemorrhage and adverse events. Using this model, we examined the cost-effectiveness of ITP treatment in which rituximab, for which clinical trials are underway to support approval for this indication in Japan, is added to splenectomy and the TPO-RA romiplostim, which are currently indicated for the treatment of ITP in Japan. The treatment costs associated with ITP treatment have been reported mainly for IVIG and splenectomy. However, there are few reports that investigated the cost-effectiveness of ITP treatment with a Markov model. In the reports that investigated the cost-effectiveness of ITP treatment, the cost-effectiveness of IVIG was compared with that of corticosteroids [23], or the cost-effectiveness of standard treatment including rituximab was compared with that of a combination of standard treatment and romiplostim [9,11].

The following points can be highlighted as unique to our analyses. First, this study examined the cost-effectiveness of only 3 treatments: splenectomy, romiplostim, and rituximab. In the Japanese guidelines, danazol, azathioprine, and cyclophosphamide are recommended as third-line treatment options, in addition to TPO-RAs and rituximab. However, we considered that the American guidelines for ITP [5] recommend only splenectomy, TPO-RAs and rituximab as second-line treatment options, and TPO-RAs represent the only third-line treatment option covered by medical insurance in the Japanese guidelines. In addition, clinical trials of rituximab are underway to support the approval of the drug for treating ITP in Japan. Second, in previous studies, effectiveness was defined as achieving a platelet count ≥50 × 10^9^/L, whereas in this study, the evaluation endpoint was set as the number of years with a platelet count ≥30 × 10^9^/L. Because rituximab is recommended in the American guidelines for ITP [5] when the platelet count falls below 30 × 10^9^/L, which also increases the risk of death or hemorrhage, we considered that the endpoint should be set as a platelet count of 30 × 10^9^/L. Third, several previous studies only considered hemorrhage as an adverse event, whereas we evaluated the adverse events that are expected to have a significant effect on the cost-effectiveness in each treatment based on specialists’ opinions and the literature. Fourth, in the application data of romiplostim for NICE, the study was conducted on the premise that the efficacy of romiplostim differs before and after splenectomy [9,10]. However, the mechanism of a difference in the efficacy of romiplostim before and after splenectomy is not clear, and this difference may arise from the different durations of disease prior to the start of romiplostim treatment; thus, in this study, the analysis was conducted assuming that the efficacy of romiplostim was the same before and after splenectomy.

This study demonstrated that adding rituximab to splenectomy-romiplostim (sequence 1) reduces the treatment cost over a 2-year period, extends the period with a platelet count ≥30 × 10^9^/L, reduces the number of deaths, and reduces the number of patients manifesting adverse events as well as those requiring emergency treatment (Table 3). The reductions in the numbers of deaths, patients manifesting adverse events, and patients requiring emergency treatment is probably because the shortened length of time for the platelet count falls to <30 × 10^9^/L by adding rituximab compared to those of splenectomy and romiplostim alone. The reduction in cost is largely related to the fact that although some patients relapsed, patients who achieved a complete cure and required no further treatment were by no means rare.

The application data for eltrombopag for NICE indicated that treatment in the order of rituximab → eltrombopag → romiplostim → IVIG is cost-effective [10]. Mowatt et al. reported that the administration of rituximab prior to standard treatment is cost-effective [9]. This information is consistent with the results of this study, in which the cost-effectiveness ratio of splenectomy-rituximab-romiplostim (sequence 3) was lower than that of splenectomy- romiplostim-rituximab (sequence 2). However, the numbers of deaths and adverse events for sequence 2 were lower than those for sequence 3. Therefore, further long-term investigation using quality-adjusted life years (QALY) is needed to determine whether sequence 2 or 3 is more cost-effective.

The results of the sensitivity analysis illustrated that the efficacy of splenectomy had the most significant effect on the difference in the cost-effectiveness ratio between sequences 1 and 2. This is probably because the reduction in the efficacy of splenectomy increases the number of subjects who move to maintenance therapy, which results in an increased number of patients who require emergency treatment in sequence 1. The efficacy of splenectomy and the romiplostim dose significantly affected the difference in cost-effectiveness ratios between sequences 1 and 3. Regarding the romiplostim dose, it is considered that more subjects were administered romiplostim in sequence 1 than in sequence 3, which had a marked effect on the cost because of the increased romiplostim dose and resulted in a larger difference in the cost-effectiveness ratios between the sequences. It is possible that the mortality rate, even if the platelet count is <30 × 10^9^/L, is no longer 4.2-fold higher than that of the healthy population because the referred mortality rate was determined prior to the availability of rituximab and romiplostim. Therefore, assuming that the mortality rate was 2.1-fold higher than that in healthy individuals, which is half of the mortality rate of the base analysis, we conducted the sensitivity analyses to assess the impact of the mortality rate. The mortality rate did not have great influence on the base case analysis.

The first limitation of this study was that we did not use utility. This is because there are no reports on utility in cases of complications with ITP or relapse, although one study used utility with the presence or absence of hemorrhage and another report mapped SF36 to SF-6D [9,10]. Therefore, we did not determine whether sequence 2 or 3 is more cost-effective in this study. We would like to accumulate data on utility for patients with ITP who developed complications. Second, the analysis period was 2 years, which did not reflect long-term drug use. Similar to this study, cost-effectiveness according to the order of treatment was analyzed in the application data to NICE for eltrombopag, and the analysis period in this study was 2 years [10]. In this study, we used a 2-year analysis period in reference to this previous study. It should be noted that although the analysis period in previous cost-effectiveness analysis of romiplostim was a lifetime, we did not use this because safety concerns such as the development of bone marrow fibrosis remains regarding the long-term use of TPO-RAs. Third, the efficacy rates of rituximab and splenectomy and the rate of relapse in this study were cited from foreign reports. However, clinical trials of rituximab are underway in Japan, and we plan to use domestic data for investigations as soon as the results become available. Fourth, eltrombopag is another TPO-RA approved in Japan in addition to romiplostim, but we selected romiplostim in this study because eltrombopag was not recommended by NICE in the UK once. The efficacy, cost, and administration methods of romiplostim are different from those of eltrombopag. Therefore, we conducted sensitivity analyses of the efficacy and cost of romiplostim. However, the results of the base case analysis were robust. Fifth, we assumed that when rituximab or splenectomy was effective, no further treatment would be undertaken. However, ITP therapeutic agents including corticosteroids are continuously administered in some cases. We also assumed that romiplostim was administered as a monotherapy, but in fact, other drugs such as corticosteroids are sometimes used in combination. In this study, however, we assumed no treatment considering the state in which the concomitant use of corticosteroids is avoided as much as possible because of its adverse events. Sixth, the actual maintenance therapy depends on a variety of factors. However, we assumed that steroid therapy would be administered as the maintenance therapy when none of the treatments such as splenectomy, romiplostim, and rituximab was effective or relapse occurred. We selected the treatment based on the relevant guidelines and specialists’ opinions.

## Conclusions

The expected costs per patient over a 2-year period for sequences 2 and 3 were lower than those for sequence 1. The expected number of years with a platelet count ≥30 × 10^9^/L was greater for sequences 2 and 3 than for sequence 1.

Based on these results, the addition of rituximab (sequences 2–3) results in lower treatment costs and greater efficacy compared to that of standard treatment for ITP in adults. According to the study results, if rituximab is reimbursed for the treatment ITP in Japan, medical expenses will be reduced.

## Abbreviations

ITP: Idiopathic thrombocytopenic purpura
TPO-RA: Thrombopoietin receptor agonist
